# Psychological uncertainty in human experience and behavior: a systematic review on measurements, induction, and effects

**DOI:** 10.3389/fpsyg.2026.1844980

**Published:** 2026-06-17

**Authors:** Mervyn Franssen, Rutger Verstegen, Pavlo Bazilinskyy, Erik D. van der Spek, Marieke H. Martens

**Affiliations:** 1 Industrial Design, Eindhoven University of Technology, Eindhoven, Netherlands; 2 Integrated Vehicle Safety, TNO, Helmond, Netherlands

**Keywords:** literature, psychological, review, systematic, uncertainty

## Abstract

Uncertainty influences our experiences, emotions, and interactions, and can play a role in the adoption of automated technologies. However, the concept of uncertainty is not clearly defined. This systematic literature review synthesizes evidence from 27 peer reviewed articles explicitly addressing the word uncertainty, on the definitions, measurements, inductions, and positive and negative effects of uncertainty in relation to human experience and behavior. Based on our analysis, we provide a definition of psychological uncertainty, which highlights the nature of psychological uncertainty as a context-dependent state, of which the effects vary per source. This review also reports on measurements of uncertainty, which most frequently are based on Likert/multiple-choice self-reporting. Furthermore, this review summarizes the different methods for inducing participants in a state of uncertainty, which are recall of uncertain events, varying or withholding information, and altering predictability. Lastly, this review reports on the moderators of uncertainty and provides one single definition of psychological uncertainty for future work to implement and refine. The results from this review can inform future studies, refine theoretical frameworks, and guide the development of practical applications in psychology and human factors.

## 1 Introduction

In an era characterized by rapid technological development and escalating reliance on automation, a nuanced understanding of how individuals perceive and respond to uncertainty has become increasingly important. The human feeling of uncertainty influences our experiences, emotions, and interactions in multiple ways. Uncertainty is related to experiencing negative emotions (Anderson et al., 2019; Morriss et al., 2022; Franssen et al., 2024b). Influencing the perception of this construct is the intolerance of uncertainty, which is a trait that reflects negative beliefs about uncertainty and its effects (Koerner and Dugas, 2008). Human behavior is shaped by experiences of uncertainty. For example, in research on driving behavior, higher levels of uncertainty have been shown to impact lane-change decision-making, leading to increased reaction times (Yan et al., 2023). This indicates that uncertainty can impede timely and accurate human responses. Lee and See (2004) discuss uncertainty's negative effect on trust in technology, which in turn has a negative impact on the acceptance and adoption of emerging and automated technologies.

In daily circumstances, uncertainty is also expected to play a role. For example, in daily traffic, people may be uncertain about travel times or other details of their travel. Uncertainty about other people's behavior and lack of anticipation can decrease safety (Mühl et al., 2020). Although recent road safety statistics indicate a modest decline in traffic-related fatalities, the numbers remain alarmingly high (WHO, 2023), with high societal costs (Bougna et al., 2022). Vehicle automation has often been proposed as a potential means of reducing such accidents (Jeong et al., 2017). However, while automated vehicles (AVs) may (partially) remove the role of uncertain drivers of vehicles, other road users may still experience uncertainty regarding a vehicle's intentions, behavior, and dependability.

Automation, as mentioned above, refers to technology capable of autonomously collecting data, interpreting information, making decisions, and controlling system actions (Beller et al., 2013). As automation becomes more advanced, it is fundamentally transforming how other road users and passengers interact with their vehicles (Krueger et al., 2016; Dong et al., 2024; Mandujano-Granillo et al., 2024; Izquierdo et al., 2025; Zhang et al., 2021). It is important to understand and respond appropriately to uncertainty that often arises from a lack of knowledge and information during interaction between humans and automated systems (Meissner et al., 2020; Lingam et al., 2025a), to adequately create clear, intuitive, and unambiguous interaction between these automated systems and humans. The presence of uncertainty can negatively influence trust (Lingam et al., 2026) and therefore have an impact on the adoption of new (automated) technologies (Lee and See, 2004). Studies show that people can struggle to form accurate mental models of the capabilities and limitations of automated driving systems (Seppelt and Lee, 2007; Nasser et al., 2025), a potentially indirect indication of psychological uncertainty. Consequently, drivers may feel unsure about the behavior, reliability, and underlying intentions of these systems (Beller et al., 2013). Psychological uncertainty can influence emotional states (Morriss et al., 2022; Franssen et al., 2024a) and is known to affect human behavior (Windschitl and Wells, 1996). It may therefore play a key role in how passengers and other road users experience and interact with AVs.

Uncertainty has been researched in a broad context due to the polysemous nature of the term uncertainty. Recent work has stated that reviewing the literature on uncertainty is highly challenging, given the breadth of the concept and the frequent conceptual overlap among related terms (Gu et al., 2020). Scholars have repeatedly noted that “uncertainty” does not refer to a single, unified construct but rather encompasses multiple meanings depending on the theoretical perspective and domain of application (Milliken, 1987; Han et al., 2011). Uncertainty can be experienced in many forms (e.g., environmental uncertainty, cognitive uncertainty, psychological uncertainty, model uncertainty, state uncertainty, etc.). Cognitive uncertainty is uncertainty within mental representations and reasoning (Pouget et al., 2016; Meyniel et al., 2015) and overlaps the most with psychological uncertainty. Psychological uncertainty includes the mental experience of uncertainty and combines this with cognition, emotion, motivation, and behavioral output (Morriss et al., 2022; Franssen et al., 2024b; Anderson et al., 2019).

Psychological uncertainty has been researched in the field of AVs in the work of Franssen et al. (2024a) and can be measured subjectively. In the work of Franssen et al. (2024a), psychological uncertainty was measured on a scale from “absolutely not uncertain” to “absolutely uncertain” in the context of AV interior design. Lingam et al. (2025b) uses a similar approach with different wording, using the phrases: “no uncertainty” to “absolute uncertainty,” to measure psychological uncertainty in human drone interactions.

In the field of judgment and decision-making (JDM), uncertainty is commonly treated as an epistemic feature of probabilistic structures, referring to situations in which outcomes are not fully determined, and probabilities are less than certain. By Savage (1954), this concept is formalized by subjective probability, when a person has a belief of what may happen with probabilities above 0 and below 1. Kahneman and Tversky (1979) reconceptualized decisions under uncertainty as a psychological process, which can be characterized by biases such as over- and underestimation of chance. Although JDM research occasionally addresses the emotional consequences associated with uncertain situations (e.g., stress, discomfort, or anxiety; see e.g., Camerer and Weber, 1992), these are typically regarded as responses to epistemic uncertainty rather than as an experiential quality of uncertainty itself. In the present literature review, we focus on psychological uncertainty. Our usage of this term builds upon the aforementioned notions that uncertainty arises as a result of subjective probabilities, and that such uncertainty can have affective consequences. However, to avoid conflating our use of uncertainty with the JDM perspective, we make an explicit distinction. Rather than treating uncertainty solely as an epistemic characteristic of the decision environment, we conceptualize psychological uncertainty as its experiential manifestation. Accordingly, we conceptualize psychological uncertainty as an experiential state in the minds of individuals, which can become salient through perceptions of the situation or environment and perceptions of one's own internal state.

Terms that may seem similar but are distinct from uncertainty are uncontrollability, unpredictability, and ambiguity. Compared to unpredictability and uncontrollability, the term uncertainty is broader and can refer to any kind of unknown (Gu et al., 2020). Ambiguity, on the other hand, refers more specifically to something being perceived as equivocal or as insufficient information to permit a singular, definitive interpretation (Gu et al., 2020; Carleton et al., 2012).

Psychological uncertainty is often not or poorly defined, and even if the construct is defined, its definition varies between studies (Anderson et al., 2019). Literature often relates psychological uncertainty back to other grounded constructs, such as trust (Lee and See, 2004), and negative valence emotions (Morriss et al., 2022; Franssen et al., 2024b), but lacks a clear definition of what psychological uncertainty is.

Literature in the context of AVs and uncertainty demonstrates the varieties of ways that uncertainty is used. The concept is used in a multitude of contexts, such as the modeling of uncertainty in algorithms (e.g., Mirzabagheri et al., 2025) or communicating the uncertainty of AVs to other road users (e.g., Luo et al., 2025). Concerning human uncertainty, the work of Jayaraman et al. (2018) discusses uncertainty reduction and research crossing types and driving behavior and their effects on pedestrian crossing. Notable publications in the field of AVs that focus on the psychological state of uncertainty are Mühl et al. (2020), Franssen et al. (2024a), and Yan et al. (2023). With these examples, we demonstrate that there is work that focuses on psychological uncertainty. However, the scope of such work is often narrow, there is often a lack of focus on how uncertainty is defined and its effects on human behavior. Therefore, this literature review aims to broaden the concept of uncertainty by moving beyond the field of human factors and AVs to shed light on the definitions, measurements, and effects of psychological uncertainty in the existing body of literature. Accordingly, we pose the following research questions (RQs):

- **RQ1:** How can psychological uncertainty be defined?
- **RQ2:** How can psychological uncertainty be measured?
- **RQ3:** How can a state of psychological uncertainty be induced?
- **RQ4:** What negative effects can psychological uncertainty have on humans?
- **RQ5:** What positive effects can psychological uncertainty have on humans?

Our systematic literature review is constructed to conclude a clear definition of psychological uncertainty that can be used for research in the fields of human factors and AV design. The present review is deliberately bounded in scope. Rather than aiming to provide a comprehensive synthesis of all constructs related to uncertainty in psychology, we focus specifically on studies that explicitly mention uncertainty as a human experience and uncertainty related to human behavior. In this review, we use the term psychological uncertainty to refer specifically to experiential uncertainty, that is, uncertainty as it is subjectively experienced by individuals. From this perspective, subjective probabilities and confidence judgments are considered part of psychological uncertainty only insofar as they are experienced as states of uncertainty. As such, closely related constructs are not systematically reviewed unless they are explicitly framed as instances of uncertainty itself. This decision reflects a conceptual and methodological choice to examine how “uncertainty” is operationalized, induced, and studied when it is treated as an explicit psychological construct. The final definition will expand upon the work of Lingam et al. (2025a) which uses Windschitl and Wells (1996) as a basis for a definition of general uncertainty: “*Uncertainty, a critical human factors challenge, ‘is assumed to be an important mediator in situations (interactions) with unknown outcomes (in the minds of humans)”'*. Furthermore, this article makes two contributions to the field of psychological uncertainty: (1) it provides an overview of the current state of research, and (2) it identifies and formulates knowledge gaps for human factors research in the context of AVs. The insights from this review can inform future studies, refine theoretical frameworks, and guide the development of practical applications in psychology and human factors.

## 2 Methods

**Figure 1:**
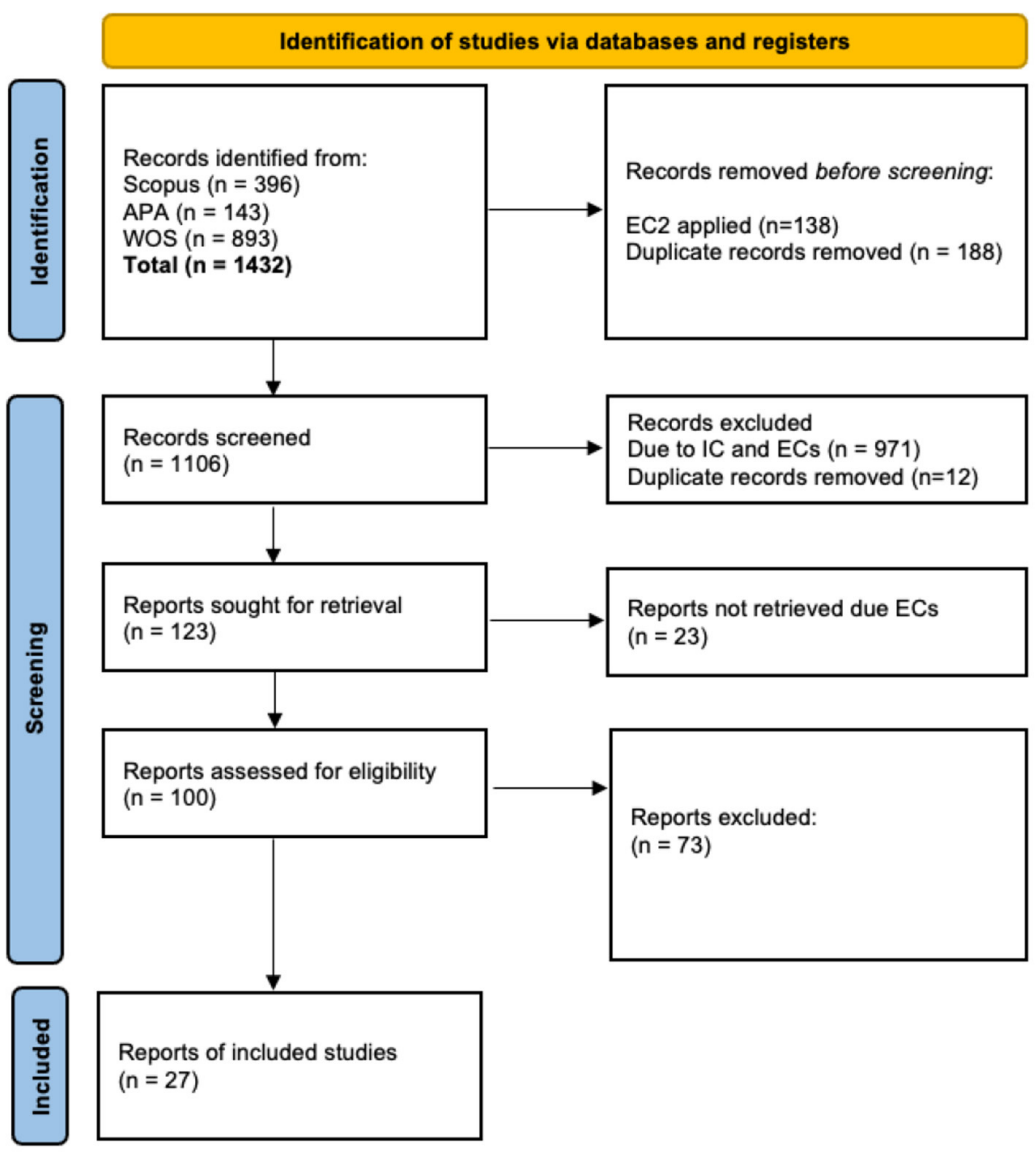
Overview of the literature review search process. Filled out template based on the PRISMA standards (Page et al., 2021), Licensed under CC BY 4.0 (https://creativecommons.org/licenses/by/4.0/).

The present article presents a critical review of the current state of research on psychological uncertainty and identifies existing gaps in the literature. The study followed a systematic literature review approach, guided by the PRISMA 2020 framework (Page et al., 2021), to ensure transparency and replicability. Boolean operators, limits, and filters were fully reported according to PRISMA-S (Rethlefsen et al., 2021) guidance. The selection process included a structured search across multiple databases with academic studies, applying predefined inclusion and exclusion criteria. Relevant studies were analyzed to synthesize key definitions, measurement approaches, and report the effects of psychological uncertainty. For a graphical overview of the process, see Figure 1. For the purpose of reproducibility and open science, supplementary material of the process followed in this publication is made available.

### 2.1 Search databases

Our search was performed in three databases: Scopus, Web of Science (WoS), and the APA PsycArticles database (which was accessed via ProQuest^1^). These three databases were selected for this review based on their focus on human factors, behavior, and psychology.

### 2.2 Search queries and filters

To identify relevant publications, we have iteratively created a search query that combines relevant search terms and their synonyms related to psychological uncertainty. Due to broad usage of the term uncertainty, early pilot searches with search queries focusing on uncertainty yielded over 3,500 results. To scope down the review, the authors aimed to identify what overlap existed in publications that did not conceptualize uncertainty as a mental experience. Therefore, the following publications that contained these search terms have been excluded to ensure that the scope of the search stayed within the boundaries of human factors. The search strategy included exclusion terms to reduce the retrieval of literature outside the predefined scope. This caused search terms like “object”, “economic,” and “clinical” to be excluded. Furthermore, “object” was excluded because object uncertainty mainly concerns incorrect object measurements in physics. “Economic” was excluded because economic uncertainty can refer to a situation where individuals, businesses, and governments find it difficult to predict future economic conditions (Baker et al., 2016), which makes decision-making harder, and decision-making theory falls outside of the scope. Lastly, “clinical” was excluded because clinical uncertainty refers to a situation in healthcare where a clinician does not have complete or definitive information to determine the best diagnosis, prognosis, or treatment for a patient (Wray and Loo, 2015), which also falls outside of the scope. “AI,” “animal,” and “conspiracy” were later also excluded to further narrow the scope of the study to only look at the human feeling of uncertainty. We do not limit the results to automation-related uncertainty; we aim to include all work that defines or measures psychological uncertainty to synthesize a coherent definition of psychological uncertainty for human factors research (e.g., in the field of automation). Through inclusion and exclusion criteria, this study aims to explore the uncertainty positioned in the context of automation interactions. Through the IC, ECs, and search query, the review covers only literature that explicitly labels its focal construct as “uncertainty”. For each iteration of the query, we reflected if the amount of identified records was manageable to review and if it reflected the reports that are interesting for this literature review, as done in similar work (Lorenz et al., 2024).

The query was adapted to fit the three selected databases (the specific queries for each database can be found in the supplementary material). If possible in the database, search filters were applied to include conference and journal publications. An example query can be found below.

TITLE (“Uncertaintyc”) ALL (“psychology” OR “attitude” OR “psychological” OR “perceived” OR “subjective” OR “feeling” AND NOT “ai” AND NOT “economic” AND NOT “animal” AND NOT “object” AND NOT “conspiracy” AND NOT “clinical”) KEY (“Uncertainty”) AND (LIMIT-TO (PUBSTAGE, “final”)) AND (LIMIT-TO (SUBJAREA, “PSYC”)) AND (LIMIT-TO (DOCTYPE, “ar”) OR LIMIT-TO (DOCTYPE, “ch”) OR LIMIT-TO (DOCTYPE, “cp”)) AND (LIMIT-TO (LANGUAGE, “English”)).

### 2.3 Search results

The search was performed on 12 March 2025, and yielded *n* = 1,432 identified records (Scopus = 396, WoS = 893, APA = 143). The search was conducted, and the results were exported to a CSV file on 12 March 2025. Entries with missing data were removed (e.g., missing abstracts or keywords), as this may have caused incorrect processing during filtering or query steps (e.g., failure to exclude reports lacking keywords). Next to that, duplicate records were removed. These steps led to the exclusion of *n* = 326 records, leaving a total of *n* = 1,106.

### 2.4 Independent screening

To grade whether the records met the inclusion and exclusion criteria, all titles and abstracts were screened independently by two raters using Microsoft Excel. During this stage, data that were not relevant for the judgment of the inclusion criteria (e.g., publication year, citation amount, and authors) were omitted, so that the authors would focus only on the content of the publications. The independent review was conducted using two separate files, ensuring that each rater remained blind to the other's assessments. Records were rated ordinally, allowing the raters to indicate if they strongly argued for rejection, argued for rejection, felt neutral, argued for acceptance, or strongly argued for acceptance. For each record, the raters noted the rationale for their rating. The screening involved the following inclusion and exclusion criteria.

#### 2.4.1 Inclusion criteria (IC)

- **IC:** The study concerns the experience or definition of psychological uncertainty, perceived uncertainty, or the feeling of uncertainty in the context of human behavior, as its main topic, or it directly correlates it with the main topic.

#### 2.4.2 Exclusion criteria (EC)

- **EC1:** Must be a peer-reviewed full article (e.g., work-in-progress and short articles excluded).
- **EC2:** Database must at least provide title, abstract, keywords, and full text.
- **EC3:** Full text must be accessible through institutional rights of Eindhoven University of Technology or Open Access^2^ and in English.
- **EC4:** Abstract must contain the word ‘uncertainty'.

Following the independent screenings, the raters' outcomes were systematically compared. This grading was performed with a 60% agreement and a quadratically weighted Cohen's Kappa of κ=0.36, indicating a ‘fair' agreement (Landis and Koch, 1977). This ‘fair' agreement can be explained due to uncertainty being a polysemous term used differently across disciplines, as discussed in the introduction. This notion is further highlighted by Gu et al. (2020), who called reviewing all the work on uncertainty and arriving at a conclusion ‘tremendously hard' due to the confusion between similar concepts and the breadth of the concept. Due to this difficulty, interpretation from the raters was required on whether the usage of the term ‘uncertainty' truly reflected the IC. The inter-rater reliability between the raters is expected to be ‘fair' due to this need for interpretation rather than strict observation. Screening based on abstracts was experienced by the raters as challenging due to ambiguity in how uncertainty was operationalized and variation in the extent to which it constituted a central focus of the study. To illustrate this, two disagreements are exemplified below:

*Report 1022:* ***R1:*** “*Main focus on information processing flow. This does not fit the IC.” (Verdict: Reject)* ***R2:*** “*Based on the abstract, it is unclear if the study focused on the experienced uncertainty or not.” (Verdict: Neutral)*.

*Report 1002:* ***R1:*** “*Link between decision-making abilities and uncertainty tolerance is examined.” (Verdict: Accept)* ***R2:*** “*Paper focuses on uncertainty tolerance, but not on the actual experience of uncertainty itself. Therefore, it does not meet the IC.” (Verdict: Reject)*.

Disagreements were discussed by the two raters, who went over the record and the related motivations for the performed grading. After all disagreements were solved, this led to the inclusion of the final 123 reports that were taken to the next step of retrieval.

### 2.5 Full report categorization

From the 123 reports sought for retrieval, 23 reports were not retrieved due to meeting the exclusion criteria. All full-text PDFs of the publications that were retrieved were again reviewed independently in Microsoft Excel based on the inclusion and exclusion criteria, similar to the methods used for the first screening. This was done with a quadratically weighted Cohen's Kappa of κ = 0.38, again indicating a “fair” agreement, with an agreement of 66% (Landis and Koch, 1977), which could be explained by the interpretations required by the polysemous usage of uncertainty. To further examine the inter-rater agreement, we reviewed the 44 instances of initial disagreement. None of the disagreements involved classifications at the extreme ends of the scale (e.g., “strongly argue accept” or “strongly argue reject”), further illustrating that disagreements reflected minor differences in interpretation regarding the fit with the IC rather than substantive differences in screening judgments. This is further illustrated with an example:

*Report 694:* ***R1**: “Main focus on brief temporal relations between micro-processes. Study looks more into how this influences experienced uncertainty than vice-versa.” (Verdict: Reject)* ***R2**: “Paper relates utterances of uncertainty and disagreement. The way uncertainty is defined and is scoped, the paper is a border case on if it meets the IC. There is some focus on the perceived elements in the introduction, but the analysis uses purely linguistics” (Verdict: Neutral)*.

760: ***R1**: “Main focus and conclusion on moral uncertainty and not being able to be fully certain on a moral. This does not fit the IC” (Reject)* ***R2**:“Paper researches differences between empirical and normative uncertainty. I consider the direct link to be (barely) sufficiently but investigation into different types of uncertainties are interesting” (Verdict: Accept)*.

Again, the differences in outcomes were solved based on common agreement taking into account details of the records and the rationales of the raters, which led to the inclusion of the final 27 publications.

### 2.6 Data extraction

After the screening phase, the 27 included research articles were then analyzed to elicit data for answering the research questions of this publication. Relevant parts of the publications were selected, and an overview of their contents was made in Microsoft Excel. This process was split based on the research questions between the first two authors. Both authors were required to read all publications. No snowballing was applied, meaning that references that were cited and articles that cited the 27 research articles were not analyzed. The results were cross-verified, meaning that the first two authors reviewed each other's work. An overview of the included publications and the research questions that each publication contributed to can be found in Table 1. More specifically, all definitions of uncertainty were extracted verbatim from the included studies (into the Microsoft Excel), together with any cited sources. These definitions were independently examined and inductively compared by both first authors, after which the work was cross-verified and discussed jointly to resolve discrepancies. This process consistently identified two dimensions: a lack of information or knowledge and an inability to predict outcomes or events, which were used to structure the definition matrix (Table 2). Similarly, induction types, measurements, and the framing of positive/negative effects of uncertainty were inductively compared and cross-verified. Disagreements between the authors were documented and all were resolved through discussion based on full-text review. Lastly, the studies were rated on their quality based on their methodological robustness and inferential strength, which can be found in Table 3.

**Table 1:** Overview of included articles and their meta-data.

Article	Publication year	Citation count	Author country	Definition of uncertainty	Is uncertainty measured?	Uncertainty framed positively	Uncertainty framed negatively	Is uncertainty induced?
Whitecross and Smithson (2023)	2023	15	Australia	No	No	Yes	Yes	No
Dağtas and Sehnaz Sahinkarakas (2024)	2024	5	Turkey	Yes	‘Self-report: Multiple choice/Likert'. (Ambiguity on if the other methods were used to measure uncertainty as well)	Yes	Yes	No
Alquist et al. (2020)	2020	15	USA, Australia	Yes	‘Self-report: Multiple choice/Likert'	No	Yes	Varying or withholding information
Kupor et al. (2014)	2014	37	USA	No	No	No	No	Recall of uncertain events
Montoya et al. (2015)	2015	17	USA	No	No	No	No	Varying or withholding information
Alison et al. (2015)	2015	53	UK	No	Other	No	Yes	Other
Chumakovaa and Kornilovb (2013)	2013	32	Russia	No	No	No	No	No
Rios et al. (2014)	2014	22	USA	No	No	Yes	No	Recall of uncertain events
Kurtz et al. (2007)	2007	133	USA	No	No	Yes	No	Varying or withholding information
Greco and Roger (2003)	2003	492	UK	No	No	No	Yes	Altering predictability
Howell (1971)	1971	116	USA	No	No	No	No	Other
Smith et al. (2007)	2007	200	Australia, USA, UK	No	‘Self-report: Multiple choice/Likert' (Study 2)	No	No	Recall of uncertain events
Bar-Anan et al. (2009)	2009	612	USA	Yes	No	Yes	No	Other
Mullin and Hogg (1999)	1999	228	Australia	No	‘Self-report: Multiple choice/Likert'	No	No	Other
Sankaran et al. (2023)	2023	8	India, Poland, UK	No	Self-report: other	No	No	Altering predictability
Salah et al. (2023)	2023	48	Tunisia, USA	Yes	‘Self-report: Multiple choice/Likert'	No	Yes	No
Hirsh et al. (2012)	2012	942	Canada, UK	No	No	No	No	No
Alquist and Baumeister (2024)	2024	1	USA, Germany	Yes	No	Yes	Yes	No
Terashima and Takai (2019)	2017	4	Japan	No	No	No	Yes	Recall of uncertain events
van Horen and Millet (2022)	2022	3	Netherlands	Yes	‘Self-report: Multiple choice/Likert'	No	No	Recall of uncertain events
Gregory et al. (2021)	2021	2	USA, Spain	No	Study 1: ‘Self-report: Other' (Other studies more ambiguous)	Yes	No	Varying or withholding information
Grupe and Nitschke (2011)	2011	179	USA	No	No	No	Yes	Altering predictability
Lamnina and Chase (2019)	2019	188	USA	No	‘Self-report: Multiple choice/Likert'	Yes	No	Varying or withholding information
Wu et al. (2022)	2022	15	China	Yes	‘Self-report: Multiple choice/Likert'	Yes	Yes	Recall of uncertain events
Speekenbrink (2022)	2022	25	UK	No	No	Yes	No	No
Junça-Silva and Caetano (2023)	2023	10	Portugal	Yes	No (Measurement specific for work environments only so not applicable)	No	Yes	No
Anseel and Lievens (2007)	2007	93	Belgium	No	No (Measurement too specific)	No	No	No

**Table 2:** Definition matrix.

Article	Definition of uncertainty	Lacking info or knowledge	Inability to predict
Dağtas and Sehnaz Sahinkarakas (2024)	“The subjective feeling of doubt and not knowing how to decode the present, the future, and the past (Jordan and McDaniel, 2014)”. “In the present study, which is part of a comprehensive Ph.D. research, uncertainty is described as a psychological, personal, subjective experience of not being certain and not being able to make an accurate prediction concerning individual aspects, other people, and the features of the context.”	Yes	Yes
Alquist et al. (2020)	“Uncertainty involves an individual lacking important information (Bar-Anan et al., 2009).”	Yes	No
Bar-Anan et al. (2009)	“Uncertainty refers to the state of an organism that lacks information about whether, where, when, how, or why an event has occurred or will occur (Knight, 1921).”	Yes	Yes
Salah et al. (2023)	Page 1: “Uncertainty reflects a lack of confidence in one's ability to predict particular outcomes (Penrod, 2001), and it may exact a significant burden on emotional and mental wellbeing (Eastwood et al., 2008; Eche et al., 2019; Perez et al., 2020).”	No	Yes
Alquist and Baumeister (2024)	Page 1: “Uncertainty is a cognitive-emotional state of not knowing which of multiple possibilities is or will be true (FeldmanHall and Shenhav, 2019).”	Yes	Yes
van Horen and Millet (2022)	Page 2: “Uncertainty due to external, unpredictable events (e.g., pandemics, economic crises) is typically characterized by a combination of aleatory and epistemic uncertainty. Because of the complexity of the events and the absence of reliable estimates regarding their occurrence and outcomes, people are not able to make an assessment of the probability that, for instance, their job will be lost due to an economic crisis (Faraji-Rad and Pham, 2017; Milliken, 1987). We therefore define uncertainty as the inability to estimate the impact of unpredictable societal or personal events on one's life, and the incapability to predict their associated outcomes.”	No	Yes
Wu et al. (2022)	Page 1: “Uncertainty is defined as ‘when a person confronts an inability to predict the future or an incompatibility between different cognitions, between cognitions and experiences, or between cognitions and behavior' (van den Bos and Lind, 2002).”	No	Yes
Junça-Silva and Caetano (2023)	Present on page 1: “The COVID–19 pandemic crisis has increased the levels of uncertainty defined as an experience of insecurity about the unknown (Hillen et al., 2017)- due to the diverse social, economic, and organizational changes (Deev and Plíhal, 2022; Junça-Silva and Silva, 2022).” | Also present on page 2: “Uncertainty “is fundamentally a mental state, a subjective, cognitive experience of human beings rather than a feature of the objective, material world. The specific focus of this experience, furthermore, is ignorance–i.e., the lack of knowledge. “(Anderson et al., 2019).”–Present on page 2: “Uncertainty can be defined as an awareness of the unknown (Anderson et al., 2019), however, even when an individual is aware of what s/he does not know, s/he tends to feel discomfort and anxiety, as individuals, in nature, prefer what they know and what is predictable (due to their need for control).”	Yes	No

**Table 3:** Evidentiary rate assessment.

Article	Methodological robustness	Rationale: methodological robustness	Inferential strength	Rationale: inferential strength
Whitecross and Smithson (2023)	Moderate	Online survey study n = 398 using relevant measures. Due to online study using self-report we give moderate robustness.	High	Conclusion fits the evidence well.
Dağtas and Sehnaz Sahinkarakas (2024)	Moderate	Only one class of n = 23, but *in situ* research using multiple measures.	Moderate	Study theoretically suggested a model but this is not validated.
Alquist et al. (2020)	High	Three studies with 50, 92, and 51 participants with well-structured procedure.	High	Strong counterfactual logic.
Kupor et al. (2014)	High	Strong procedure with relevant measurements.	Moderate	Conclusions are fitting the data. Results on low tolerance varied by study and implications on other domains would need to be researched.
Montoya et al. (2015)	Moderate	Two studies with relevant measures but smaller samples. Procedure is briefly described making study harder to interpret.	Moderate	Discussion is related back to previous literature, but studies require follow-up research for potential explanations of results offered in discussion.
Alison et al. (2015)	Moderate	Small sample size but relevant measures and interesting experiential procedure.	Moderate	Strong typological inference, however no experimental manipulation check confirming uncertainty.
Chumakovaa and Kornilovb (2013)	High	Good sample, with relevant measured and strong analysis.	Moderate	Results are strong and conclusion is correct but claims regarding behavior of other people could be further elaborated in the discussion. Cultural generalizability is not addressed.
Rios et al. (2014)	High	5 studies researching creativity self-uncertainty and even general uncertainty using a strong procedure.	High	Bridges domains: Integrates uncertainty, identity threat, and creativity. Conclusion is based well on results.
Kurtz et al. (2007)	Moderate	2 studies, well-described but generally small sample size and brief in execution.	Moderate	Strong claims that suit the data, but should be interpreted with caution given the limited study and its specific scope.
Greco and Roger (2003)	Moderate	One study with student sample. Short time-frame study.	Moderate	Health implication are diluted from theoretical work. Direct implications are therefore not made towards health. Furthermore, behavioral implications are not discussed.
Howell (1971)	Moderate	Interesting methodology, but small sample size and short study. Was interpreted in respect of time given the age of the publication.	Moderate	Establishes a foundational claim (but study is limited to support this claim broadly) and cleanly isolates sources of uncertainty.
Smith et al. (2007)	High	Multi study design with clear procedure, measures and proper analysis.	Moderate	Behavioral outcomes are indirect. Mechanisms are theorized, but not tested.
Bar-Anan et al. (2009)	Moderate	Multi study design. Little differentiation between studies 1-3. Overall procedure is well-described.	Moderate	Study offers explanations for results in study 1-3 but these would require additional testing. Overall results are well-based on the results but explanations for the results sometimes required further grounding.
Mullin and Hogg (1999)	Moderate	Multiple theoretically diagnostic dependent variables. Manipulation checks. Laboratory setting with small groups.	Moderate	Outcomes are motivational and attitudinal rather than behavioral. Mechanisms are inferred rather than manipulated.
Sankaran et al. (2023)	Moderate	Two studies with sufficient participant groups and relevant measures.	Moderate	Need for closure is measured, not manipulated. Does not show whether heightened perceived control actually reduces uncertainty post-task.
Salah et al. (2023)	Moderate	Big representative sample. Questionnaire study that measured though a single item uncertainty performed during covid-19.	Moderate	Study is performed during covid-19, raising questions on generalizability. Also, it is heavily dependent on self-reporting.
Hirsh et al. (2012)	Moderate	No own study, but conceptualization of new ideas. Setup would have required validation/test for high robustness.	Moderate	Multiple directions of literature are combined, but the framework requires additional validation for support.
Alquist and Baumeister (2024)	Moderate	No own studies in this article. Research provocation using existing works to suggest a positive view on uncertainty. Used work is limited.	Moderate	Sharp and clear interpretation of literature that combines literature from multiple fields and offers research directions, but given the specific work used more follow-up studies are required, this is in line with the claims of the authors.
Terashima and Takai (2019)	Moderate	Multi study publication showing three studies with undergraduate samples. Clear methods: Did induce uncertainty, but did not measure uncertainty.	Moderate	Conclusions reasonably based on results, but further validation of behavioral implications required.
van Horen and Millet (2022)	High	Methods are very well documented and structured reasonably. Multiple studies with good samples. Methods were clear and uncertainty was induced and measured.	High	Results are clearly based on findings with real world relevance. Overall, mechanism have been specified and tested well.
Gregory et al. (2021)	High	Multi study experiment, partially pre-registered with *in situ* testing and relevant measures throughout the experiments.	High	Field evidence confirms real-world behavioral relevance, not just reflective attitudes. The article demonstrates when, why, and how uncertainty leads to enhanced positive emotion regulation across correlational, experimental, and behavioral levels.
Grupe and Nitschke (2011)	Moderate	Smaller study that alterated predictability and did not measure uncertainty directly. Well performed and documented.	High	Study nuances its claims well and targets its research intentions. Strong theoretical integrations.
Lamnina and Chase (2019)	High	Well performed and documented study with 12 school classes. Induction check was used and study utilized well grounded research artifacts and relevant measures.	Moderate	Demonstrates that uncertainty can be pedagogically productive when managed, rather than avoided. Learning outcomes did not differ by condition, tempering claims about uncertainty enhancing all forms of learning.
Wu et al. (2022)	High	Three study approach (study 1 has study 1a and 1b) with good sample sizes, relevant measures, and solid analysis.	Moderate	Uncertainty reliably increases present-oriented choice, leading individuals to prefer smaller-sooner rewards across contexts and measures. Focuses on direct outcomes; does not test downstream behavioral domains.
Speekenbrink (2022)	Moderate	Paper ties together Bayesian literature with other pieces of literature to discuss uncertainty. No own study with human participants.	Moderate	Paper advances strong, coherent theoretical claims, but it does not introduce new empirical tests or falsify competing explanations within the article itself, limiting how decisively its conclusions can be established.
Junça-Silva and Caetano (2023)	High	Paper features two studies from both telecommuters (during confinement) and non-telecommuters. Overall, the study was well structured, featured good participants, and had good analysis. Appropriate multilevel modeling, reliability checks, and robustness replication across contexts.	Moderate	Although the article uses rigorous multilevel diary designs and replication across two samples, its conclusions are limited to correlational and mediated pathways that cannot establish strong causal direction or rule out alternative explanations. Uncertainty operationalization through the used measurements limits the implications of the findings for this particular literature review.
Anseel and Lievens (2007)	Moderate	Two studies with sufficient participants samples. Methods are heavily dependent on self-report and could be further clarified.	Moderate	The paper strongly refines theory and rules out simple linear accounts, but does not fully establish causal mechanisms. The measure of uncertainty was checked, and the operationalization of the measure was too specific for this literature review, making the implications of the findings less relevant for this particular review.

### 2.7 Meta-data extraction

Metadata were extracted and analyzed following the final screening phase. Given the limited sample of 27 included articles, the aim of the metadata extraction is descriptive, characterizing the identified body of work rather than providing a comprehensive bibliographic overview of the field. The included articles were geographically mapped according to the countries listed in the affiliations of all contributing authors. For each article, each country was counted once; that is, multiple authors from the same country within a single article contributed a single country count (e.g., an article with three authors from the United States and one from Greece is counted as having contributors from both the United States and from Greece). Additionally, the citation count for each article was retrieved from Google Scholar on 26 September 2025. These data were collected to create a transparent understanding of how the impact of research countries, publication years, and citations varied among the included articles.

## 3 Findings

### 3.1 Details of the identified publications

**Figure 2:**
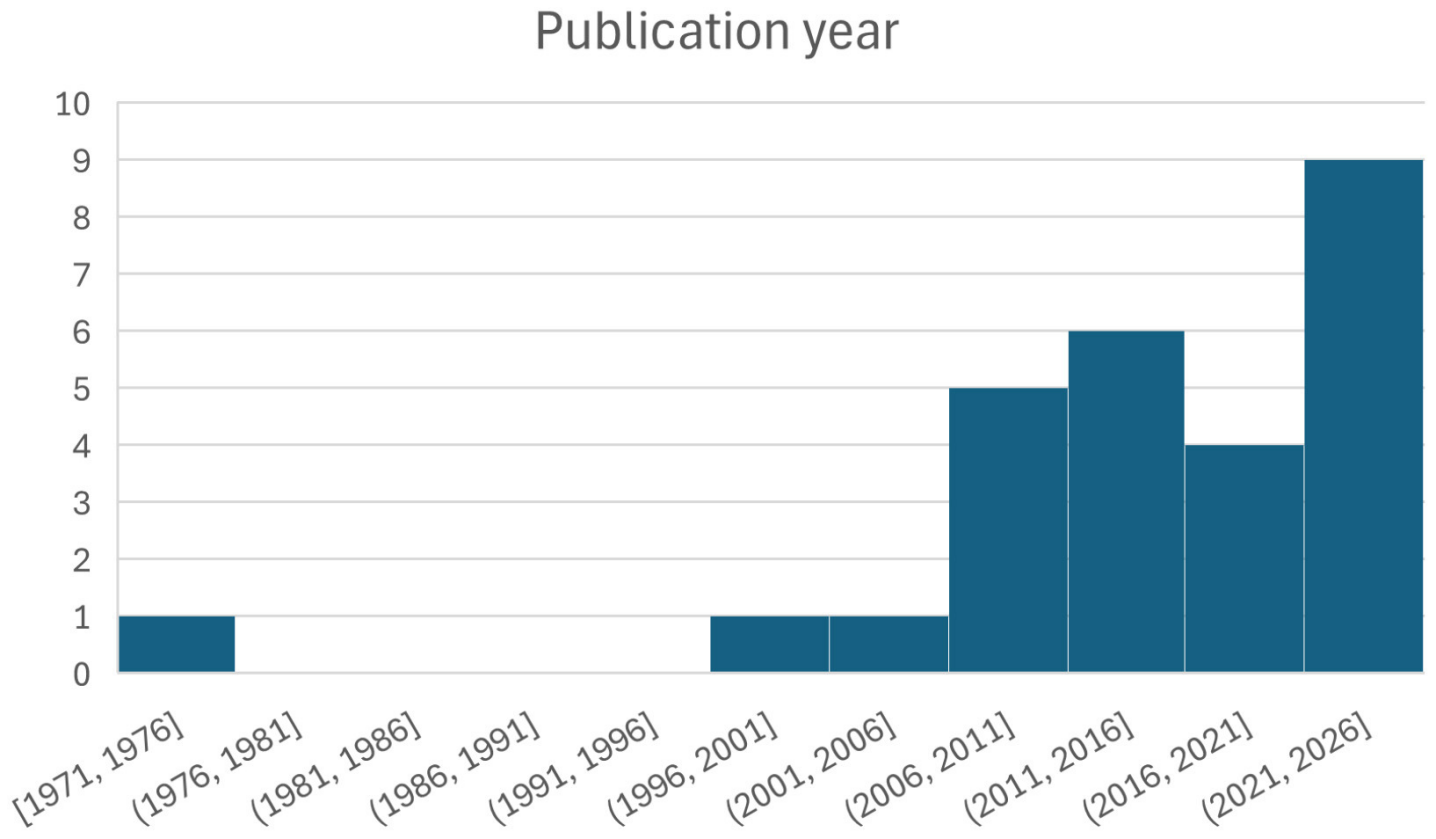
Graph of publication years.

**Figure 3:**
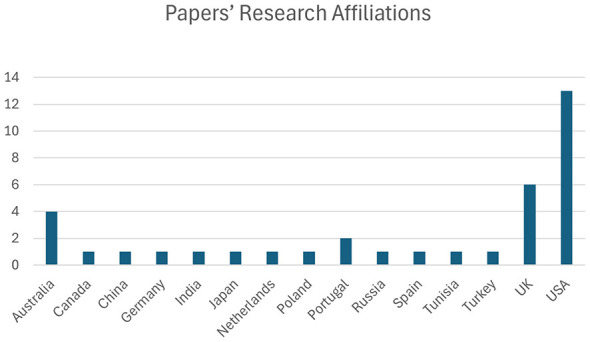
Graph of articles' research affiliations.

**Figure 4:**
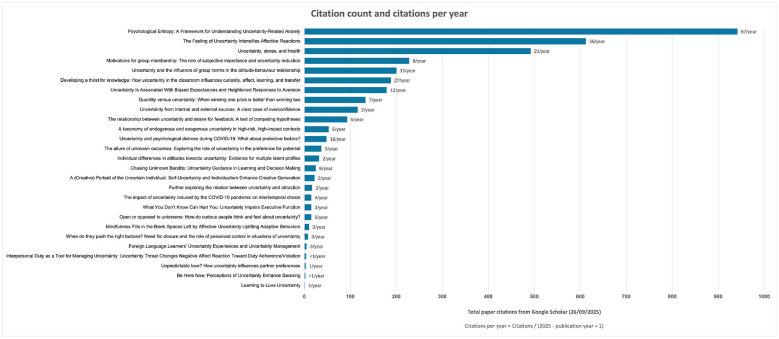
Graph of citation count (Google Scholar) 09-26-2025.

The final 27 publications demonstrate an increase in research on uncertainty, as can be seen in Figure 2. With 24 out of 27 works being published/printed past 2006, and 2021-2026 being the time frame with the most (9) publications. Research affiliations are primarily from the USA (13), UK (6), and Australia (4), see Figure 3. To understand the academic impact of the identified work, both the citation count and citations per year are plotted in Figure 4. As shown, the three works that garnered the most citations are Hirsh et al. (2012); Bar-Anan et al. (2009), and Greco and Roger (2003), with the article of (Hirsh et al. 2012) leading. Considering citations per year, again the work by Hirsh et al. (2012) and Bar-Anan et al. (2009) is in the top three, with the third place being occupied by Lamnina and Chase (2019).

### 3.2 RQ1: definitions of psychological uncertainty

To gain insights into definitions of psychological uncertainty, our review analyzed if the 27 selected articles included a formal definition of what uncertainty was in the context of their publication. For this purpose, brief statements focused on describing the uncertainty were accepted as definitions, whereas 8 articles included a definition of uncertainty (Dağtas and Sehnaz Sahinkarakas, 2024; Alquist et al., 2020; Bar-Anan et al., 2009; Salah et al., 2023; Alquist and Baumeister, 2024; van Horen and Millet, 2022; Wu et al., 2022; Junça-Silva and Caetano, 2023). A definition matrix was made showing all 8 definitions, their references and their overlap, as can be seen in Table 2. An examination of the definitions of uncertainty revealed that all definitions were based on references to other publications (Dağtas and Sehnaz Sahinkarakas, 2024; Alquist et al., 2020; Bar-Anan et al., 2009; Salah et al., 2023; Alquist and Baumeister, 2024; Wu et al., 2022; Junça-Silva and Caetano, 2023). From the articles that included a definition of uncertainty, 3 definitions referred to the property of lacking information or knowledge (Alquist et al., 2020; Bar-Anan et al., 2009; Junça-Silva and Caetano, 2023). Next to that, three definitions focused on the inability to predict the future/outcomes (Salah et al., 2023; van Horen and Millet, 2022; Wu et al., 2022). Closely related, uncertainty was defined by Alquist and Baumeister (2024) as not knowing what will be true from multiple possibilities and by Dağtas and Sehnaz Sahinkarakas (2024) as a “subjective feeling of doubt and not knowing how to decode the present, the future, and the past”. Despite the overlap in the definitions used for uncertainty, a check of the referenced work for these definitions shows that all definitions reference different existing work, with the exception of the article by Alquist et al. (2020) referring to the definition used in the article by Bar-Anan et al. (2009).

### 3.3 RQ2: measurements of uncertainty

The reviewed literature shows that 11 articles included measurements of psychological uncertainty (Dağtas and Sehnaz Sahinkarakas, 2024; Alquist et al., 2020; Mullin and Hogg, 1999; Salah et al., 2023; van Horen and Millet, 2022; Wu et al., 2022; Smith et al., 2007; Lamnina and Chase, 2019; Sankaran et al., 2023; Gregory et al., 2021; Alison et al., 2015). Publications that contained measures which did not focus on psychological uncertainty in a manner general enough to be transferable to human factors (e.g., Greco and Roger, 2003 who used the Uncertainty Response-Emotional (UR-E) from the Uncertainty Response Scale by Greco and Roger (2001), or Anseel and Lievens (2007) who used the Self-Attributes Questionnaire by Pelham and Swann (1989) which measured uncertainty about traits) were therefore excluded from this section.

Analysis of the 11 measurements of perceived uncertainty shows that uncertainty was primarily measured in the form of self-reports using either multiple choice or Likert scale questions (Dağtas and Sehnaz Sahinkarakas, 2024; Alquist et al., 2020; Mullin and Hogg, 1999; Salah et al., 2023; van Horen and Millet, 2022; Wu et al., 2022; Smith et al., 2007; Lamnina and Chase, 2019). Similar methods for self-reporting were drawing a line on a scale (Sankaran et al., 2023) and moving a slider between 0–100 (Gregory et al., 2021). Next to self-reporting, Alison et al. (2015) used observational methods to examine verbal and written expressions of uncertainty.

### 3.4 RQ3: induction of uncertainty

For the purpose of our review, we analyzed how participants were induced into states of uncertainty. Uncertainty was most commonly induced through 3 different ways:

1. Recall of uncertain events.
2. Varying or withholding information.
3. Altering predictability of study conditions.

#### 3.4.1 Recall of uncertain events

In order to gain insight regarding humans being in an uncertain state, many studies induced participants to experience uncertainty. A frequently used method was by asking participants to recall or write about (past) uncertain events (Kupor et al., 2014; Terashima and Takai, 2019; van Horen and Millet, 2022; Wu et al., 2022), an aspect in life that made them uncertain (Rios et al., 2014), or an unresolved personal dilemma (Smith et al., 2007).

An example of such an uncertainty manipulation can be found in the work of van Horen and Millet (2022), who mentions the following manipulation in the supplementary material:

“*Imagine yourself in a situation where you felt uncertain. Use your imagination and write a short story about that event, indicating in detail what happened, how you felt, and how strong the impact was on your personal life. Take 2-3 minutes (max. 100 words) to complete the essay and then proceed with the study. Use the following words in your essay: ‘uncertain' – ‘unclear' – ‘insecure' – ‘unsure' – ‘unpredictable'.”*

#### 3.4.2 Varying or withholding information

Also frequently used for inducing a state of uncertainty was the method of varying the information or instructions that were given to participants. For example, by providing a group of participants with more ambiguous instructions on whether they would need to give a speech or not (Alquist et al., 2020), or by providing instructions at different moments in the assignment (Lamnina and Chase, 2019). Other studies manipulated uncertainty by withholding information, such as a lack of knowledge on how their Facebook profile was rated (Montoya et al., 2015) or what prize they won (Kurtz et al., 2007). Studies also intended to provide participants with a different framing of uncertainty (or a lack thereof) presented, for instance, via advertisements (Kupor et al., 2014) or in video and flier material (Gregory et al., 2021).

#### 3.4.3 Altering predictability of study conditions

Some works altered probability or predictability, for instance, by not providing cues for predictability (Greco and Roger, 2003) or explanations regarding cues, the meaning of cues (Grupe and Nitschke, 2011), or changing the probability of a certain outcome after an action (Sankaran et al., 2023).

#### 3.4.4 Other methods of uncertainty induction

Next to the first three ways of inducing uncertainty, other strategies for inducing uncertainty were making participants repeat phrases that conveyed uncertainty (Bar-Anan et al., 2009) and altering external vs. internal components of uncertainty using a probability spinner vs. dart throwing, respectively (Howell, 1971). Notably, one study performed a live hostage negotiation simulation (Alison et al., 2015). In the study of Mullin and Hogg (1999), task uncertainty was provoked by telling participants that some views on a subject can actually be more correct than those of others due to better factual grounding.

### 3.5 RQ4: negative effects of uncertainty

From all 27 included publications, 11 concluded and discussed that uncertainty had negative effects on their participants (Whitecross and Smithson, 2023; Dağtas and Sehnaz Sahinkarakas, 2024; Alquist et al., 2020; Alison et al., 2015; Greco and Roger, 2003; Salah et al., 2023; Alquist and Baumeister, 2024; Terashima and Takai, 2019; Grupe and Nitschke, 2011; Wu et al., 2022; Junça-Silva and Caetano, 2023). Uncertainty was consistently associated with adverse affective, cognitive, motivational, and social outcomes in these studies.

#### 3.5.1 Discomfort, affective strain, and stress-related responses

Uncertainty is associated with discomfort stemming from the feeling of not “knowing” (Whitecross and Smithson, 2023). The work of Greco and Roger (2003) expanded upon the knowledge of discomfort caused by uncertainty by proving that the prospect of facing an unknown, potentially threatening situation can lead to discomfort and cause health implications. Physiologically, the prospect of unknown and potentially threatening circumstances elicited stress responses reflected through cardiovascular reactivity, a pattern relevant to stress-related disorders and downstream health risks (Greco and Roger, 2003).

In population-level contexts (e.g., the COVID-19 pandemic), perceived uncertainty correlated with symptoms of depression and anxiety, though these associations were mitigated by social support and psychological resilience (Salah et al., 2023). The work of Grupe and Nitschke (2011) showed that uncertainty amplifies the negative impact of aversive events, at both physiological and behavioral levels; repeated exposure may foster an association between the state of uncertainty itself and aversion. In the context of educational activities, participants in the work of Dağtas and Sehnaz Sahinkarakas (2024) reported negative appraisals and feelings, along with reduced motivation and unwillingness to participate; the most frequently observed emotions were boredom, stress, nervousness, sadness, and restlessness as a reaction to their perceived uncertainty and the discomfort thereof. Daily work performance also appeared sensitive to uncertainty: in office-based samples, uncertainty increased negative affect and, in turn, reduced adaptive performance (Junça-Silva and Caetano, 2023). Heightened affective sensitivity to duty adherence and violation, together with greater changes in negative affect, is experienced under uncertainty (Terashima and Takai, 2019). The work of Terashima and Takai (2019) indicated that in the context of social unrest or personal crises, such sensitivity was linked to mutual criticism and hostility.

#### 3.5.2 Decision making

Uncertainty impairs decision quality both directly (by complicating evaluations of competing options) and indirectly, by inducing behavior consistent with cognitive fatigue (Alquist et al., 2020). The work of Alison et al. (2015) found that when making decisions, uncertainty may occur as a product of endogenous sources (e.g., those specific to the context of the decision environment) or as a result of exogenous sources (e.g., those caused by problems with the operating system responding to the decision problem). Endogenous uncertainty within choice environments was identified as a driver of heightened uncertainty, derailment of choice implementation, and decision inertia (Alison et al., 2015). Lastly, uncertainty causes a present-focused orientation, increasing satisfaction with immediate gains and encouraging short-sighted decision tendencies, a pattern that can manifest as greater impatience and preference for immediacy over longer-term outcomes (Wu et al., 2022).

### 3.6 RQ5: positive effects of uncertainty

The reviewed literature also related uncertainty to positive effects. From the 27 selected publications, 10 concluded and discussed that uncertainty had positive effects on their participants (Kurtz et al., 2007; Bar-Anan et al., 2009; Dağtas and Sehnaz Sahinkarakas, 2024; Alquist and Baumeister, 2024; Whitecross and Smithson, 2023; Gregory et al., 2021; Wu et al., 2022; Speekenbrink, 2022; Lamnina and Chase, 2019; Rios et al., 2014). Uncertainty was consistently associated with positive affective, cognitive, motivational, and social outcomes in these studies. Recurring themes mentioned the findings of studies related to the experience of positive feelings, creativity, learning, and attention.

#### 3.6.1 Extending pleasure and positive feelings

The experience of uncertainty is shown to be able to increase the duration of the feeling of pleasure in case of positive outcomes, such as winning unknown prizes (Kurtz et al., 2007). Next to extending the duration, it can lead to more intense affective reactions toward positive events (Bar-Anan et al., 2009). Lastly, needing to deal with uncertain situations can develop positive emotions (Dağtas and Sehnaz Sahinkarakas, 2024; Alquist and Baumeister, 2024). As mentioned in the work of Whitecross and Smithson (2023), uncertainty can lead to pleasure from speculation of outcomes and from the positive anticipation of learning something.

Related to the experience of positive emotions, feelings of uncertainty can lead to savoring of the present moment (Gregory et al., 2021). It has also been shown to be linked with more satiation with immediate gains, though these can also be related to short-sightedness (Wu et al., 2022).

#### 3.6.2 Learning, attention, and curiosity

Multiple publications mentioned positive effects of uncertainty related to learning and attention (Alquist and Baumeister, 2024; Speekenbrink, 2022). In their research regarding science instructions, (Lamnina and Chase, 2019) found that uncertainty can increase the experienced curiosity, which then can allow for greater transfer of knowledge to other contexts. The work by Whitecross and Smithson (2023) further nuances the relationship between uncertainty and two types of curiosity: deprivation and interest curiosity. Their findings show a link between deprivation curiosity (exploration to relief from the state of not knowing) and the intolerance of uncertainty and a focus on negative possibilities, whereas interest curiosity (related to enjoyment due to anticipating learning new knowledge) was associated with experiencing uncertainty as more enjoyable.

Research by Rios et al. (2014) found that creative performance can be elicited most strongly by uncertainty about the self compared to uncertainty in general, particularly when creative tasks can be seen as an option to restore the sense of self. In their work, Alquist and Baumeister (2024) discuss that introducing uncertainty can increase engagement due to outcomes still being open, making participants put in effort for a specific outcome.

### 3.7 Individual differences: influence of intolerance of uncertainty

Some works present the potential positive influence of uncertainty on humans as hinging upon how intolerant humans are of uncertainty. Findings by Whitecross and Smithson (2023) show how people who are more tolerant toward uncertainty relate uncertainty to interest and positive outcome expectancies, whereas intolerance of uncertainty relates to deprivation and negative outcome expectancies. Dağtas and Sehnaz Sahinkarakas (2024) further discuss how those who appraised uncertainty as encouraging and challenging led to satisfied and happy feelings, whereas negative appraisals were linked with negative feelings. The research by Kupor et al. (2014) shows that in conditions of moderate interest to individuals, those in the high tolerance of uncertainty condition were inclined to prefer potential gains.

## 4 Discussion

### 4.1 On the definitions of psychological uncertainty (RQ1)

There were only 8 articles that provided a definition of uncertainty, as seen in Section 3.2. Although these given definitions showed overlap in one or multiple ways, almost all articles used unique related work to build these definitions upon. Based upon these definitions and the negative and positive effects of psychological uncertainty, we propose that “*Psychological uncertainty is the mental experience of a lack of knowledge about the past, present, or future, which makes it impossible to accurately predict outcomes or determine which of multiple possibilities will be true, and which in turn shapes emotional responses and behavioral output.”* This definition represents a conceptually informed starting point for articulating psychological uncertainty and is intended to serve as a foundation for future research, rather than as a final or exhaustive account.

### 4.2 On the measurements of uncertainty (RQ2)

Across studies, uncertainty is most often assessed via self-report using multiple-choice items or Likert-type scales (Dağtas and Sehnaz Sahinkarakas, 2024; Alquist et al., 2020; Mullin and Hogg, 1999; Salah et al., 2023; van Horen and Millet, 2022; Wu et al., 2022; Smith et al., 2007; Lamnina and Chase, 2019) which is in line with the works of Franssen et al. (2024a) and Franssen et al. (2024b), showing the possibility of measuring uncertainty toward automation using a Likert scale format. Variants include visual analog lines (Sankaran et al., 2023) and continuous sliders (0–100) (Gregory et al., 2021), which is also used in the work of Lingam et al. (2025b), also contributing toward the validation of uncertainty measurement within the subject of automation.

Notably, only the study of Alison et al. (2015) moved beyond the application of self-reporting, since they used observational coding of verbal and written expressions of uncertainty. Greco and Roger (2003), tested for the correlation between cardiovascular reactivity and uncertainty, but did not find a significant relationship. Based on these results, additional research on additional objective (e.g., cardiovascular reactivity) and subjective (e.g., indirect physiological or behavior indicators) measurement methods of psychological uncertainty may be beneficial to further advance the measurability of this construct in diverse contexts. Such measurement methods can further increase the application of psychological uncertainty without relying on users actively reflecting and reporting their uncertainty, which could be useful in situations that require a user's attention on other tasks.

### 4.3 On the induction of uncertainty (RQ3)

To study uncertainty, researchers used various experimental inductions. A frequently used approach is episodic recall/writing, asking participants to reflect on past uncertain events (Kupor et al., 2014; Terashima and Takai, 2019; van Horen and Millet, 2022; Wu et al., 2022), life aspects that elicit uncertainty (Rios et al., 2014), or unresolved personal dilemmas (Smith et al., 2007). Another common strategy manipulates information availability or instructional clarity—keeping participants waiting, providing ambiguous instructions (Alquist et al., 2020), or varying when guidance is delivered during tasks (Lamnina and Chase, 2019). Studies also withhold information, such as feedback on social evaluation (e.g., unknown Facebook ratings) (Montoya et al., 2015) or prize identity (Kurtz et al., 2007). Framing manipulations present uncertainty in ads (Kupor et al., 2014) or multimedia materials (Gregory et al., 2021).

Other inductions alter predictability/probability, by removing predictive cues (Greco and Roger, 2003), obscuring cue meaning (Grupe and Nitschke, 2011), or changing outcome probabilities after actions (Sankaran et al., 2023). More exclusive methods include repetition of uncertainty-laden phrases (Bar-Anan et al., 2009), differentiating external vs. internal uncertainty via probability spinner vs. dart throwing (Howell, 1971), and a live hostage negotiation simulation to embed uncertainty within complex social coordination (Alison et al., 2015). Mullin and Hogg (1999) primes epistemic ambiguity (e.g., asserting that certain views can be “more correct” than others). All inductions focus on creating a state of experienced limited knowledge that prevents the participant from forming an accurate understanding of the past, present, or future, making it impossible to predict outcomes and to determine which of multiple possibilities will be true.

The clustering of these methods for inducing participants into a state of uncertainty revealed three strategies: recall of uncertain events, varying or withholding information, and altering the predictability of study conditions. Below, we discuss the differences between these methods, which merit further research. Unlike the methods that rely on changing information or probabilities, the recall-based approach relies on past experiences with uncertainty. Inherent to this method is the lack of control over what experiences participants draw upon. This lower level of control may introduce variability between participants and studies. Simultaneously, we wish to stipulate the potential of this approach for gaining richer insights into potentially heterogeneous properties of the experiences of uncertainty. On the other hand, approaches that focus on changes in information or probabilistic structures offer more consistency in the conditions of participants and may therefore still be favorable for studies that require higher levels of control, such as those in lab environments.

Given that the definition of uncertainty proposed earlier in this discussion relates uncertainty to not being able to predict outcomes accurately, we wish to refute that changes in predictability lead to uncertainty in a direct manner. Specifically, altering predictability is no guarantee of participants truly experiencing uncertainty, since participants might incorrectly feel overly certain. Therefore, we argue that studies focusing on perceived uncertainty should always perform induction checks to verify if subjects truly experienced the intended perceived uncertainty.

Closely related to induction checks, we consider that different induction methods may lead to different experiences of uncertainty. For instance, a change in predictability of a lab condition, such as the blinking of a light, may induce a different experience compared to the recall of a personal experience, potentially impacting past experiences of uncertainty.

Observing the identified articles and the studies performed, we notice a lack of naturalistic/field studies focusing on the occurrence of uncertainty in real-world settings. Such studies could further deepen our understanding of uncertainty by, for instance, investigating factors fostering (or reducing) the experience of perceived uncertainty, as well as naturalistic behavioral and (physiological) expressions of uncertainty.

### 4.4 Effects of appraisals and moderators of uncertainty

The effects of uncertainty can depend on its appraisals, its moderators, the type of uncertainty, or on the situation that it is present in. Rather than uncertainty yielding inherently positive or negative outcomes, the results demonstrated more nuanced and context-dependent effects. Accordingly, the present discussion intentionally considers both potential benefits and costs of uncertainty as they emerge under different appraisals and conditions. Whether uncertainty harms or helps often hinges on intolerance of uncertainty and evaluation. Higher tolerance is related to interest and positive outcome expectations; higher intolerance is related to deprivation and negative expectations (Whitecross and Smithson, 2023). The assessment of uncertainty as encouraging/challenging predicts satisfied and happy feelings, whereas negative assessments predict negative affect (Dağtas and Sehnaz Sahinkarakas, 2024). Under moderate interest and high tolerance, individuals are more inclined to prefer potential (uncertain) gains, suggesting a Goldilocks zone where uncertainty motivates rather than paralyzes (Kupor et al., 2014). Endogenous uncertainty (seen as uncertainty about the problem/task itself) can derail choice implementation, while exogenous uncertainty (system-level unreliability) undermines trust and coordination (Alison et al., 2015). The stakes, controllability, and time horizon shape whether uncertainty is assessed as an opportunity or threat, adjusting the affective and cognitive profile accordingly (Wu et al., 2022; Terashima and Takai, 2019). By outlining the conditions associated with both positive and negative effects of uncertainty, the present article emphasizes that its consequences are context dependent.

#### 4.4.1 Negative effects of uncertainty under threat appraisals

Negative effects of uncertainty can occur when it is appraised as a lack of control or threatening. Uncertainty causes affective strain through the discomfort of “not knowing” (Whitecross and Smithson, 2023). That discomfort is physiologically instantiated: the prospect of unknown, potentially threatening circumstances elicits cardiovascular reactivity, linking uncertainty to stress-related pathways and downstream health risks (Greco and Roger, 2003). Psychologically, uncertainty can therefore also be linked to a state of not knowing. In population-level crises (e.g., COVID-19), perceived uncertainty correlates with depressive and anxiety symptoms, whose effects can be mitigated by social support and psychological resilience (Salah et al., 2023), showing the fluid nature of psychological uncertainty and the possibility to alter the amount of experienced psychological uncertainty based on the context. Uncertainty can amplify the impact of aversive events and, via repeated exposure, become conditioned as an aversive state in its own right (Grupe and Nitschke, 2011). In educational settings, learners who had a negative attribution to uncertainty associated it with boredom, nervousness, and restlessness (Dağtas and Sehnaz Sahinkarakas, 2024). In office-based samples, elevated daily uncertainty increases negative affect and reduces adaptive performance, while mindfulness dampens these negative effects (Junça-Silva and Caetano, 2023). Social dynamics are similarly vulnerable: heightened sensitivity to duty adherence/violation under uncertainty is linked to mutual criticism and hostility, especially amid social unrest or personal crises (Terashima and Takai, 2019). This link between negative effects and negative side effects goes hand in hand with the importance of mitigating psychological uncertainty once it negatively impacts a user's day-to-day behavior and mindset, and therefore avoids people negatively reacting to others, prompting them to experience an increased amount of psychological uncertainty as well.

In decision-making, uncertainty impairs evaluations directly and indirectly via cognitive fatigue (Alquist et al., 2020). Endogenous uncertainty within choice environments (ambiguous criteria, volatile parameters) drives derailment and decision inertia; exogenous uncertainty (system unreliability) further degrades implementation quality (Alison et al., 2015). Uncertainty also promotes a present-focused orientation, increasing satisfaction with immediate gains and encouraging short-sighted choices (impatience, immediacy preference) over longer-term outcomes (Wu et al., 2022). Decision-making is crucial in interaction with autonomous systems; users should not feel uncertain about the actions that they are required to make. Psychological uncertainty should not prompt users to make short-sighted decisions and therefore misuse a system or cause states of regret in later usage.

#### 4.4.2 Positive effects of uncertainty through opportunistic appraisals

When appraised positively, however, uncertainty can lead to positive outcomes. In favorable contexts, it extends the duration of pleasure (e.g., winning unknown prizes) and intensifies affective reactions to positive events (Kurtz et al., 2007; Bar-Anan et al., 2009). Dealing with uncertain situations has been linked to the development of positive emotions (Dağtas and Sehnaz Sahinkarakas, 2024; Alquist and Baumeister, 2024). Feelings of uncertainty can also foster savoring of the present, enhancing appreciation of immediate experiences, even as present focus may also risk myopia (Gregory et al., 2021; Wu et al., 2022). Fostering positive emotions to create a positive feeling through psychological uncertainty can be a key element in why calibration of psychological uncertainty in the context of interaction with automated systems can be beneficial. Influencing the amount of psychological uncertainty to create as much positive affect to induce a good interaction is as important as ensuring that psychological uncertainty is not over-experienced, possibly leading to overstimulation and overwhelm.

Under self-uncertainty, participants can be more engaged and absorbed in creative work, and calibrated uncertainty can increase participation and interest (Alquist and Baumeister, 2024). In learning and attention, uncertainty can heighten curiosity and facilitate knowledge transfer, particularly when instruction harnesses uncertainty productively (e.g., inquiry, hypothesis testing with scaffolds and timely feedback) (Alquist and Baumeister, 2024; Speekenbrink, 2022; Lamnina and Chase, 2019). The key here is that these exists in the context of self-uncertainty, which in and of itself does not have to be a positive state or experience. It is therefore important to balance the positive experiences if they are caused through negative experiences.

### 4.5 Downstream implications for automation

Motivated by advancements in automation, this literature review explored studies that explicitly focused on psychological uncertainty in human experience and behavior. This section situates the findings in the context of automation. The scope of this literature research was purposefully broadened beyond the field of automation to expand upon the possible relations between uncertainty and its related constructs to find downstream implications for automation.

Firstly, this publication operationalized a definition for psychological uncertainty and generated an overview of measurement methods of uncertainty (RQ1&2). Both contributions can be directly applied to future studies in the field of automation. The overview gained from researching the inductions of uncertainty (RQ3) demonstrates a lack of real-time field measurements, which could be further developed and then leveraged, so that these could be applied by AVs.

By researching the positive and negative effects of psychological uncertainty (RQ4&5), we provide a nuanced view on the topic, which deviates from the (often) critical positioning of uncertainty that is present in the context of vehicle automation (Yan et al., 2023; Mühl et al., 2020). Instead, we highlighted that the effects of uncertainty are context dependent rather than inherently positive or negative. Further, by positioning the identified positive and negative effects that uncertainty can have on behavior, we wish to highlight the complex nature of the consequences of psychological uncertainty. For instance, one might argue that the intensification of affective reactions toward positive events (Kurtz et al., 2007; Bar-Anan et al., 2009) may make participation in traffic more pleasurable, whereas others could argue that stronger affective reactions can take away focus from participating in traffic or secondary tasks in AVs. Similarly, the experience of discomfort that can be introduced by uncertainty may be bad for the mental state of those experiencing it, but may be beneficial in making traffic participants avoid potentially dangerous uncertain situations.

Based on this, we consider that, similar to trust calibration in the context of automation (de Visser et al., 2020; Walker et al., 2023), uncertainty should not be considered as something that should be completely mitigated or stimulated. Rather, we argue that some levels of certainty may be warranted in certain situations, and therefore uncertainty should be graded and compared to factual degree of uncertainty. As such, in situations that could be harmful or are not certain in their outcomes, road users should, rightfully so, experience psychological uncertainty.

However, in the context of arguing for the calibration and optimal degree of uncertainty, moderators and conditions of uncertainty should still be taken into account. For instance, the intolerance of uncertainty may to be paralyzing for users, whereas tolerance of uncertainty can lead to the preference of potential gains (Kupor et al., 2014). Therefore, we consider that future frameworks could further define how personal and situational characteristics could be leveraged for managing uncertainty in specific traffic situations (e.g., by leveraging the aforementioned paralysis in situations where this could increase safety).

### 4.6 Limitations

The chosen selection and search parameters may have led to the inadvertent omission of relevant studies, potentially narrowing the range of insights. However, these search properties were required because of the broad polysemous usage of the word uncertainty in other fields, such as those of AI and economics; this application of a broader scope risks fragmentation and broadly spread coverage, whereas this review focused on the depth of the findings. This has been noted in other research as well, which mentions that synthesizing research on uncertainty is particularly difficult due to its broad definition and the lack of clear distinctions between closely related constructs (Gu et al., 2020). Therefore, during the methodological approach, we chose to filter out reports that contained the word “object” to narrow the scope, since we noticed that object uncertainty was often related to physics articles and flooded the dataset with large numbers of articles that had to be filtered out by hand and were not realistically worth the trade-off of extensive abstract reading vs. potential gain in insights. Filtering out based on the word “object” could have also filtered out relevant articles. For example, consider the work of Bradley and Drechsler (2014), which explains different types of uncertainty and uses uncertainty on objects as the features of reality that judgments are directed at. Like the work of Lorenz et al. (2024), we have judged the exclusion terms (e.g., “economics”, “object”, etc.) based on the balance between the amount of results and relevant reports that are potentially excluded whilst being relevant, in order to create an amount of identified records that was manageable to review. To resolve the exclusion of this relevant work, we have chosen to present this and similar literature in the introduction to include relevant views on uncertainty.

The scope of this review on the experience of uncertainty and the narrow search frame used for this purpose inadvertently have effects on the implications from this review. On the one hand, to avoid conflating a complex term such as uncertainty, the findings of this study should be clearly interpreted through the authors' usage of the term uncertainty. On the other hand, whilst analyzing the identified literature, this review has demonstrated to the authors the complexities associated with the usage of a polysemous term that is used across multiple disciplines. To improve multidisciplinary communication on uncertainty, we consider that authors should explicitly specify their meaning of the term uncertainty upon usage. Similar to Anderson et al. (2019), this literature review demonstrates that the term uncertainty is often unspecified or defined in different ways. Additionally, further exploration of different disciplines and their approaches toward uncertainty could still lead to further expansion of the topic which could help better map the nuances in the usage of this term and thus facilitate improved communication.

This systematic review started from a gap in the literature on psychological uncertainty in the context of AVs in human factors. Although our search aimed to move beyond the boundaries of these fields to explore this topic, articles from this context were not explicitly excluded. The final selection of publications, however, did not contain work from the field of human factors and AVs. This could be due to this work not being retrieved due to not matching the search query or being filtered out through the inclusion or exclusion criteria. This further supports the research gap mentioned in this publication on psychological uncertainty within the field of automation. The broader search strategy applied in this review to move outside of the scope of automation may have led to findings that were harder to relate back to automation, but in perspective became suitable for the intended scope of the research. Furthermore, the strict inclusion and exclusion criteria made the pool of articles relatively small, making it more difficult to draw firm conclusions from the limited number of publications. This review takes this into account, and each argument was considered proportionally based on the volume of evidence. Furthermore, each argument mentions on which findings it is based, and the discussion complements these findings by proposing fitting suggestions for future work. The findings of this review should be generalized and interpreted considering the number of identified articles. The conclusions would benefit from further confirmation as the body of relevant articles expands. Moreover, applying these findings to the domain of AVs should be done with additional caution, given that the reviewed literature was not conducted in this context and that AVs may introduce new characteristics that may shape experiences of uncertainty differently.

### 4.7 Future work

In this article, we have proposed a single definition of psychological uncertainty: “*Psychological uncertainty is the mental experience of a lack of knowledge about the past, present, or future, which makes it impossible to accurately predict outcomes or determine which of multiple possibilities will be true, and which in turn shapes emotional responses and behavioral output.”* Although this outcome paves the way for future implementation of the research on psychological uncertainty, it would be beneficial to find the best method to measure psychological uncertainty now that it is defined. The measurement method for psychological uncertainty used by Franssen et al. (2024a), Franssen et al. (2024b), Lingam et al. (2025b), and Alquist et al. (2020), who measure on a scale from ‘not uncertain' to ‘uncertain', could be combined with induction methods like episodic recall/writing (Kupor et al., 2014; Terashima and Takai, 2019; van Horen and Millet, 2022; Wu et al., 2022; Rios et al., 2014; Smith et al., 2007), or varying when guidance is delivered during tasks (Lamnina and Chase, 2019), to further quantitatively measure psychological uncertainty and the origin thereof within complex behavior, automation adaptation and task completion. Such induction checks can already be found in the work of Alquist et al. (2020), who implemented this measurement scale whilst providing ambiguous instructions, and should be implemented in future work to confirm the presence of psychological uncertainty. Based on the different induction methods identified through this literature review, we consider that follow-up research could explore if and how psychological uncertainty differs based on the selected induction method.

Making a mental model of psychological uncertainty and finding proof of linked psychological key constructs is needed to gain a deeper understanding of how psychological uncertainty influences our day-to-day behavior and affects how we adopt the transition toward automation. Lee and See (2004) showed that uncertainty influences levels of trust, which in turn affects the acceptance and adoption of emerging and automated technologies. Future work could therefore further explore the application of real-time measurements of uncertainty, based on which AVs could adapt their behavior in real-time as well. Based on the rapid developments and the growing application of AVs, further research into the application of uncertainty for this specific context should be conducted.

## 5 Conclusion

Given a gap in the literature detailing psychological uncertainty in the context of humans interacting with automated systems, this literature review set out to explore the topic of psychological uncertainty in a systematic manner. Our analysis shows that psychological uncertainty is a context-dependent state, the effects of which vary per source, like endogenous ambiguity, exogenous unreliability, appraisal, stakes, controllability, individual differences, etc. Negative effects caused by psychological uncertainty include discomfort and stress-related physiological activation, amplified aversive reactions, dampened motivation and engagement, and impaired decision quality. However, when psychological uncertainty is calibrated and evaluated as a challenge under manageable stakes, it can extend and intensify positive affect, cause creative engagement, and increase curiosity, attention, and learning. Therefore, psychological uncertainty does not necessarily have to be seen as a negative construct. It should be seen as a psychological state that can be calibrated to optimize human behavior.

## Footnotes

1 https://www.proquest.com/, last accessed 03-05-2026.

2 https://www.openaccess.nl/en/what-is-open-access, last accessed 03-05-2026.
